# With Our Editors

**Published:** 1907-02-15

**Authors:** 


### WITH OUR EDITORS.

**SOME THINGS LACKING IN DENTAL PROSTHESIS.**

Last month the writer considered certain advances in dental prosthetic procedures, and it would seem pertinent now to point out some imperfect methods still in use which need remedying.

It seems strange that in the many years since plaster-of-Paris superseded wax as an impression material no real improvements have been made in the fashioning of a metal base-plate. The methods of to-day are the same as they were forty years ago, which fact is certainly not a credit to the supposed inventive faculty of the average dental brain.

The plaster impression, plaster model, sand mould and zinc or Babbit-metal die with lead counter, are still the means by which we prepare to shape a piece of metal to fit an edentulous jaw and which, when supplied with artificial teeth, is intended to subserve all the intricate and difficult purposes of mastication.

All the various stages followed in the securing of the die are faulty. The plaster impression is, of course, accurate in detail, but the expansion of the plaster gives us an impression slightly larger than the part from which it was taken. A like expansion produces a model larger than the impression. The tapping of the model to insure its release from the sand gives us a mould larger even than the model. The contraction of the molten metal poured into the mould is supposed to compensate for the previous expansions, but we do not know that it does.

We have no means of ascertaining this supposed fact.

### A DIE DIRECT FROM THE IMPRESSION.

Our methods would more nearly approach exactness and the procedure be greatly simplified if there were some method by which the die could be produced direct from the impression.

The difficulty to be overcome is that of the generation of steam when a metal or alloy fusing at between 700 and 800 degrees F. is poured into an impression containing the slightest amount of moisture.

The impression material is too dense to permit the escape of the steam through its substance, and hence it has to make its way by bubbling through the molten metal, causing imperfection of the die.

Aloys fusing at or below 212 degrees F. have been employed successfully to obviate steam generation, but while producing a good reproduction they have proven too soft to resist the force required in the shaping of the plate.

Zinc and Babbit-metal have served best for the making of dies on account of their relative unchangeability under the strain of swaging, but they cannot be poured into an impression consisting in whole or in part of plaster.

### PRESSURE SWAGING.

Even if we had a method by which a perfect die could be produced direct from the impression, our present method of swaging by impact would still be faulty and unscientific. Hammer swaging is inaccurate in principle because the successive blows are never exactly in the same direction and a certain rocking occurs which seriously mars the counter, thus giving an imperfect result. Furthermore, the sudden impact of the die against the metal plate bruises or injures the fine lines on the face of the die, thus tending to lack of exactness.

Every manipulator of dental metal plates is familiar with the difference in the appearance of a die before and after swaging. The proper method would be to force the plate into conformity with the die by means of constant, exact, even pressure, through the medium of a substance less hard and more plastic than any metal.

Many presses and many substances have been experimented with, but while they have produced good results with small or partial pieces, none has yet been devised that would satisfactorily adapt a plate to the many convexities and concavities of a full denture.

We feel sure that in some other departments of mechanics the difficulty confronting us would have been overcome, but so far we have not proven equal to the task.

Perhaps if we had the perfect die and the perfect pressure method there would be less difficulty met with in the retention of metal plates and these would again come into greater favor. Unquestionably the best denture should be the one with the metal base, because of its cleanliness, its thinness, its strength and its conductivity; but the plate with the vegetable base, moulded to the model and therefore exact in detail, has literally "crowded it to the wall;" and this in spite of the many objectionable features of a vegetable base.

### PLATE RETENTION.

However, with our present methods of constructing a plate of either base, we lack the ability to produce one that will always keep its place and perform its functions under the varying conditions found in different mouths.

In what are known as "favorable cases" satisfactory fit and retention are obtained without difficulty, but in the *unfavorable* ones, with which we are all too familiar, we do not get the results we desire.

Considering the strain to which dentures are subjected in mastication, the different angles at which force is applied and the yielding character of the underlying tissues, it does seem as though we should have at command some more positive means of retention than simple adaption or atmospheric pressure.

The spiral springs of olden times were scientifically and mechanically correct in principle, and we sometimes wonder why they were allowed to fall into disuse.

Is there not some one in our profession ingenious enough to devise some mechanical means by which plates that will not keep their place by present methods can be held in position so as to serve their wearers as they need to be served, and thus add to the comfort and happiness (and possibly lengthen the lives) of the many unfortunates who no longer know the meaning of a "square meal"?

Of all those whom we are called upon to serve professionally, the ones who are most to be pitied and who stand in need of our highest skill, are those who have been deprived of their natural teeth and have failed to secure satisfactory substitutes.

Replacements of other missing parts of the human anatomy are capable of being held securely in place by mechanical means, but while it is our province to provide a more natural substitute than any other, and one that would be more truly serviceable if it were firmly secured in place, we are handicapped by conditions that have thus far not been overcome.

The world is waiting for the individual who will give us the means of satisfactory plate retention under all conditions. —S. H. Guilford, January *Stomatologist.*

**SYSTEM.** The season of the year is drawing near when it is customary to take stock and sum up the result of the year's work. It is just as important that the professional man do this as the business man. The business side of the education of the dental student has been sadly neglected in most of the dental colleges of our country. It is not only true of the dental profession, but many of the other professions as well, have been neglecting that side of the education of the student, until it has become a proverbial saying that a professional man is rarely ever a good business man. If you have never gone over your business in a systematic way before, do it this month and start in the new year knowing just what your assets and liabilities are. Then keep it up during the entire year, not in a spasmodic sort of a way, but be systematic all the time. How many times have men prominent in our profession, when the "rainy day" of advanced years or ill health came, through the lack of systematic attention to the details of their professional business, failed to provide for that day, which will surely come to all.

I have in mind some of the greatest men ever in our profession, who were supported in their later days by contributions of friends. Men who had enjoyed large incomes during their earning period, but through lack of systematic laying aside a percentage of their earnings, became in their old age subjects of charity.

While it is not possible for a professional man to accumulate great wealth by the labors of his own hands, yet by judicious investments it is possible for him to have a competency for his declining years. This may seem to some as sounding too much of commercialism, but not so, for I would be the last one to put the commercial idea as the one great thing to be attained. Yet it is imperative that we have some good *common business sense.* There have been so many strong, helpful papers read upon this subject during the past year, that it is not necessary to go into detail as to the best way to systematize one's business.

We put too large emphasis on the part that money plays in our estimate of success. The most valuable asset that a professional man has is his professional attainment and character. If there has been a good normal growth during the year, it is the condition that should maintain. In taking stock along these lines there should be searching introspection and we must be sure of our premise. This, of course, thus far is from a selfish standpoint and not from the broader view. But then, you know, it is so much easier to get men to see things from a selfish point of view than that of altruism. Work it out in detail to know your own heart in the matter. Are you serving your patient better because of better equipment, mental and physical? Or are you living a narrow sort of a grind of a life, just going over the same ground from year to year, doing the things that you think will bring you the most money? Are you traveling the little circuit of self-sufficiency and imagining that you are all there is to be taken into consideration? If so, you are probably staying at home during the session of your District, State or National Dental Association and are not giving or receiving what is due. Step right up to the glass, my boy, and take a square look at yourself and then answer *yourself*, are you satisfied?

There is another valuable asset that we too often leave off our balance sheet, and that is *health*. The care the average dentist gives to the proper condition of his body is such that all he deserves is aches and pains, while he is driving his over-taxed system to greater effort. What fools all of us are (or were), and all for a dollar. *Now*, before it is too late, is the time to begin a system of controlling our habits. We too often let them control us. You owe it to yourself and your patients as well that you keep yourself in condition to render them the best possible service. Concentration and conservation of your energy must play an important part if you are to give the *best* that is in you. You may think that you are an exception to the rule, but away down deep in your heart, *are you?*

It is a funny condition of things that nearly every man thinks that *he* is an exception to the rule, and that he can do things with impunity that are in direct violation of every law of health, but being as it is *me* (a wonderful sort of being) old mother nature will overlook it. In estimating your budget for the coming year, give a good allowance for recreation and dental societies; not forgetting that if recreation is to be of any value, it must be systematic. There should be an allowance also of time and money for some branch of study. There are but two propositions, either you are narrowing into ruts, or broadening and helping smooth out the humps and thus making the road better for someone else.

But, you say, how many appreciate the effort you put forth to add to the general good. If there be not one, do it for your own culture. It is rarely ever appreciated when the effort is made for the sake of the honor that might come to the doer.

*Strike a balance sheet,* and sum up your opportunities and possibilities and make the coming year count for a good wholesome *growth* along all lines. Which route did you say? The little branch road of selfishness, or the broad-gauged, rock-ballasted one of altruism. Yours the choice.—F. O. Hetrick, December *Western Journal.*

**A DENTAL LABOR UNION THREATENED.**

A new and startling crisis in dentistry has just been happily averted. That labor unionism could ever find a place in dentistry probably occurred to none of us, until it had almost reached the stage of actuality. The possibility is directly traceable to Metropolitan conditions, which it is to be hoped do not exist outside of New York City.

In what is termed the great "East Side" of New York there is a teeming population made up of almost exclusively foreign born, or at least foreign bred individuals. The ancestry, previous and present environments of these peoples are such that sentiment has little sway with them. Their fight is for the almighty dollar and their daily bread. In the rush for wages many find employment in dental offices—offices which are technically legal and therefor ethical, since a large part of them are not strictly advertising parlors.

These "ethical" dentists hire boys, or young men, primarily to clean the laboratory and office and to run errands. Later they watch the vulcanizer, pack "cases," polish plates, swage crowns, etc., etc., until in time they become "mechanical dentists." Not diploma men, but still they call themselves dentists—mechanical dentists.

These men are universally underpaid, comparing their work with other skilled labor. From seven to twenty-five dollars weekly is the rule, and the average is not above twelve dollars, or about two dollars a day. Many of them really excel in their work, and naturally chafe at their situation. They have accidentally acquired a handicraft which apparently leaves them no claim either to the status of a profession, or to that of an artisan. They are doing delicate and certainly skilled work for half the pay of the carpenter, the house painter, or the bricklayer. And in the Metropolis there are over three hundred men of this class.

Was not this a fertile field for the tilling of a Labor Union walking delegate? Was it strange that these men listened, when told by the labor organizers, that if they formed a Union they could compel the bosses, "the dental bosses," to pay higher wages?

There is a cant phrase that "money talks," and when you talk money and especially more money to men with families, they are apt to listen. Thus the crisis came about. At first the gatherings were small but they grew larger, and finally a meeting of all the mechanical dentists was called, and the hall was crowded with eager men. Men eager for a betterment of their conditions, but uncertain as to how best to accomplish it. Fortunately sober heads were guiding, and in the end sober judgment prevailed. Drs. Ottolengui, Ash, and Meeker were invited to address the men and they spoke in no uncertain language, giving advice which has borne good fruit.

An organization has been completed more in consonance with our regular dental associations. The society will be known as "The New York Prosthetic Dental Society." The membership and officers will be exclusively of non-graduate men who work on salary for other dentists, or else manage laboratories of their own. But in addition there is to be an

"advisory board," composed of ethical practitioners, who will help to guide this bark into safe waters. The objects of this new society are thus announced:

"It is our aim to accomplish the following: To educate ourselves by means of technical papers, clinics and demonstrations, to be given both by members and dentists from the profession at large. To start a Journal of Prosthetic Dentistry, consisting of extracts and selections from the leading dental journals; also original articles. To establish an employment bureau for the benefit of members. To endeavor to bring about the establishment in a Dental College of a night course for prosthetic dentists. Applicants to be eligible for active membership must be those who have been engaged in prosthetic dentistry for at least two years. They must be at least twenty years of age and of good moral character."

Thus it will be seen that the Labor Union project has been abandoned for the present. This does not at all mean that it has been permanently prevented. The restlessness of this large body of workers is still in existence, and unless the new organization can accomplish the task which it has undertaken, it is not difficult to guess that the other scheme may be tried.

As a Dental Labor Union, in any form, would be a disgrace to our beloved profession, it now becomes the sacred duty of the ethical practitioners of New York City to watch this new society and to give it all proper assistance, that it may succeed. The best men in our midst should promptly respond to calls for clinics, or lectures. It has been pointed out to these mechanical dentists that their only hope for a real betterment of their situation must be first through education, and secondly through an effort on their part to aid the law officers in weeding out the illegal dental assistants now doing operative work in offices of men who pretend to be legally managing their offices.

To accomplish these two ends, it may become a wise course for our colleges to establish night courses of instruction and it certainly will be well for the mechanical dentists to practically become the watch-dogs of their section to report promptly all illegal practices occurring within their district. The reason of this is patent upon a moments' reflection. They are deprived by law of the right to even take impressions, as they cannot work directly upon the patient. Often they are given miserable models, to which they fit perfect crowns or bridges which later they must re-make at personal loss, because they did not fit the mouths. With more competent men actually practicing, the work of the mechanical man must be lightened.—Dr. Ottolengui, in December 1906, *Items of Interest.*

**THE OPPORTUNITIES OF TO-DAY.**

By the time our readers receive this journal, 1906 will have followed its successors into the world of the past. An eventful year it has been, bringing much of disaster and sorrow into thousands of homes, but even more of joy and gladness into tens of thousands of other homes. While cyclones, hurricanes, earthquakes, railroad accidents have left hundreds to mourn their dead, and financial reverses have left others penniless, the year altogether has been one of unparalleled prosperity—which prosperity has never been surpassed in our country. It is no longer a question of "Shall we have good crops the coming season?" but where can we get the men to harvest them? The wheat can hardly find its way to the great centers of commerce. The farmer finds his granaries full almost to bursting, but is unable to land his crops in eastern and foreign markets for lack of facilities for moving. Those old mortgages which have so long worried and handicapped every member of the family have all been paid off and now the boys and girls can go to school and college. Those aching teeth can be attended to and a new order of things generally be brought about. Never has there been a time in our profession when things were "looking up" as much as at present. The people have money and are ready to spend it. Now is the time, if ever, for the young practitioner to locate, to become part of this great movement, to throw himself into his work with a vim and will that will convince the hard-headed farmers that there is something in dentistry and that possibly they had better see if they would not be more comfortable for a call upon the young dentist. The future is certainly full of promise to him who can see opportunities and possibilities.—Dr. J. N. Crouse, December, 1906, *Digest*.

**A DUTY TO THE PROFESSION.** If a careful examination is made of the output of our dental periodical literature it will be noted that most of the writing for the profession is done by a relatively few men compared to the total number of dentists in practice. This is probably true of every profession and yet there is no good reason why such a state of affairs should exist. In dentistry more than in most professions there is so much of a technical nature connected with our every-day work that the ingenuity of the individual is being constantly taxed to meet the demands of special cases as they arise. This develops or should develop, special thought, and this thought should be so formulated as to be readily put on paper for the benefit of the profession. It has frequently been said that every man who practices dentistry and thus avails himself of the knowledge handed down by his predecessors, or evolved by his contemporaries, is in duty bound to repay the debt by adding to the sum total of dental knowledge, and to spread this knowledge as widely as possible. If this contention is true, and we think it surely is, then every practitioner owes it to his profession to publish the results of his experience that others may benefit thereby.

It will no longer do for men to shirk behind the excuse of incompetency. All dentists are probably not qualified to write brilliant articles, but any dentist who schools his mind to the task of thinking out vividly and carefully any problem can at least write his thoughts with sufficient clearness to be understood. It is largely a matter of application and concentration. If dentists could only realize that the mere attempt to place their ideas on paper tends to clarify those ideas and make them more vivid in the mind of the writer, they would surely exhibit a greater willingness to apply themselves in this direction. It is undeniably true that the man who gets the greatest benefit from a paper is the one who writes it. This is particularly true of any paper written on a technical topic, or where original research work is necessary to bring out the salient features of the subject.

It would be well worth while for every dentist to form the habit of seating himself at his desk and describing any particular operation, or method, or line of practice which most appeals to him, even though he never intends publishing what he writes. This kind of training will develop concentration of thought and this is really the most essential thing in preparing papers which are worthy of publication. The literature of the profession should be represented by a larger list of writers and this can readily be done if more men apply themselves to the task, and bring out the latent talent which is too frequently allowed to remain dormant.—C. N. Johnson, in January *Review.*

**FAULTY EDUCATION.** Dental education is a subject which should and does interest every fair-minded and well-meaning dental teacher.. We as a profession ask the public, as well as other professions, to recognize us as among the learned professions of the world, and yet we are in many respects behind the majority of the professions and educational institutions in the absolute requirements for graduation from dental colleges, and this is due to our system of education.

I have had examination papers from the very best and from some of the very worst dental students that have come up before the State Board of Dental Examiners, and it has been my extreme privilege to review the answers given to many State Board questions. I am surprised at the brilliant and clear answers made by some, while in other cases it is extremely surprising how a student can pass through a dental college, having had the privilege of listening to pehaps only a few lectures on a subject, and yet go before a State Board and give such unheard of answers.

A question that was put by one of the State Board of Dental Examiners on the subject of anatomy was, "What muscles are brought into action in the process of deglutition?" and out of a class of thirty-two there were not more than one-third of that number who knew what deglutition meant. Another question in therapeutics asked by a dental board examiner was, "Give the treatment of mercurial stomatitis." The student's answer was, "I would wash out the stomach of the patient, give an emetic, and strychnine as a heart stimulant." And many other questions and answers quite in the same line as those just mentioned.

It seems strange that it can be possible for a student to pass through a dental college and expect to pass an examination before a State Board with so little knowledge of the subject most pertinent to his professional calling. Of course, the strictly mechanical dentist would say all that a student needs is to fill a tooth, make a band for a crown, take an impression and make a rubber plate. But in prosthetic dentistry, crown and bridge-work, the questions and answers are equally absurd. When a man stands at the chair and fills a tooth with gold, amalgam or porcelain; or takes an impression for a crown, or an artificial denture, and cannot produce on paper his method of performing such an operation, proves that there is something radically wrong with our methods of teaching, or with the student's mental capacity to grasp the explanations given in text-books or gone over in the lecture room.

If one was to make an inquiry as to who or what class of students would be most liable to make such blunders he would find that these answers are not always given by the student whose educational advantages had been limited, for many of the high school graduates are just as poor in their dental examinations as the student with a limited education when he enters the dental college—George. W. Cook, in January, 1907, *American Journal.*

